# Intratympanic versus intravenous corticosteroid treatment for sudden sensorineural hearing loss in diabetic patients: proposed study protocol for a prospective, randomized superiority trial

**DOI:** 10.1186/s13063-020-4077-x

**Published:** 2020-02-03

**Authors:** Weiqiang Yang, Xiaoling Li, Jiatao Zhong, Xueshuang Mei, Hongyu Liu, Le Yang, Liming Lu, Hongyi Hu

**Affiliations:** 1 Department of Otorhinolaryngology, Peking University Shenzhen Hospital, Shenzhen, China; 2 Hearing and Balance Function Medical Engineering Laboratory, Shenzhen, China; 3 The Eighth Affiliated Hospital of Sun Yat-sen University, Shenzhen, China; 4 Clinical Research and Data Centre, South China Research Centre for Acupuncture and Moxibustion, Medical College of Acu-Moxi and Rehabilitation, Guangzhou University of Chinese Medicine, Guangzhou, China

**Keywords:** Sudden sensorineural hearing loss, Diabetes, Randomized controlled trial, Intratympanic methylprednisolone, Intravenous methylprednisolone

## Abstract

**Background:**

Diabetes mellitus is associated with risk of sudden sensorineural hearing loss (SSNHL). Systemic and intratympanic corticosteroids are the two primary treatments for SSNHL in patients with diabetes mellitus. The benefit of intratympanic compared to systemic treatment is the reduced systemic steroid exposure and associated systemic adverse effects. Intratympanic corticosteroid administration may have potential benefits over standard systemic therapies.

**Methods/design:**

The proposed study is a prospective, randomized superiority trial. A total of 96 patients (48 in each group) will be randomized into the experimental or control group. Patients in the experimental group will receive four 1-mL doses of 40 mg/mL of methylprednisolone over a 1-week period, with a dose administered every 2 days via tympanic membrane injection into the middle ear. The control group will be administered intravenous methylprednisolone (1 mg/kg/day, maximal dose 60 mg/day) for 5 days. The primary outcome for this study is the change in hearing threshold from the first audiogram to the 30-day follow-up audiogram. Secondary outcome measures will include pure-tone average (PTA) at 90-day follow up, visual analog tinnitus scale, visual analog vertigo scale, visual analog aural fullness scale, fasting blood glucose and 2-h postprandial blood glucose during treatment, and the change in glycosylated hemoglobin (HbA1C) levels. Vital signs and otological physical examination will be performed at each follow-up visit.

**Discussion:**

The efficacy and safety of intratympanic methylprednisolone compared to intravenous methylprednisolone will be investigated in patients with diabetes mellitus and SSNHL. This trial may provide strong evidence for the efficacy and safety of intratympanic corticosteroid treatment and important clinical information for the treatment of patients with diabetes mellitus and SSNHL.

**Trial registration:**

ChiCTR, ChiCTR1800015954. Registered on 2 May 2018, Retrospectively registered, http://www.chictr.org.cn/showproj.aspx?proj=25326.

## Background

Sudden sensorineural hearing loss (SSNHL) is usually defined as a sensory neural hearing loss greater than 30 dB across three contiguous pure-tone frequencies occurring within 72 h [1]. The annual incidence of SSNHL is 27 per 100,000 in the USA [2]. Diabetes mellitus is a risk factor as well as a poor prognostic factor for SSNHL [3]. The incidence of SSNHL is 1.54-fold higher in patients who have diabetes mellitus compared to patients who do not [4]. Although the standard treatment for SSNHL is systemic corticosteroid administration, this treatment results in loss of glycemic control in patients with diabetes mellitus.

Long-term hyperglycemia in patients with diabetes mellitus may lead to microangiopathy of the inner ear, which results in sensorineural hearing loss [5]. Previous animal and human studies have demonstrated that hair cell loss is observed in the temporal bone as well as thickened and damaged capillary walls of the stria vascular in animals and humans with diabetes mellitus [6]. Several studies have demonstrated that systemic corticosteroid administration in patients with diabetes mellitus and SSNHL had no advantage in hearing improvement when compared to other treatments [7–10]. Moreover, the use of systemic corticosteroids may result in side effects such as insomnia, weight gain, gastritis, mood changes, photosensitivity, and hyperglycemia [1]. Hyperglycemia may further affect the prognosis in SSNHL and patients with diabetes mellitus may be required to increase their insulin intake [9].

An alternative treatment strategy for SSNHL is intratympanic corticosteroid administration via direct injection into the middle ear [1]. Intratympanic treatment may increase drug concentration to the target organ. Parnes et. al. demonstrated higher inner ear corticosteroids levels after intratympanic corticosteroid administration compared to systemic administration in animal models [11]. A multi-center, randomized controlled trial in 250 patients involved comparison of intratympanic versus systemic corticosteroid therapy for SSNHL: the improvements in hearing outcomes were equivalent [12]. The benefit of intratympanic compared to oral treatment is reduced systemic steroid exposure and associated systemic adverse effects [1, 12]. A non-prospective randomized controlled trial that compared systemic versus intratympanic corticosteroid treatment showed improved hearing outcomes in patients with diabetes mellitus and SSNHL.

Based on the evidence of potential advantages of intratympanic over standard systemic therapy, we will perform a randomized superiority trial comparing the efficacy of systemic versus intratympanic methylprednisolone administration in patients with diabetes mellitus and SSNHL. The results of the study may provide evidence to determine whether intratympanic corticosteroid administration is a better primary treatment option for patients with diabetes mellitus and SSNHL.

## Method/design

### Design and setting

**Fig. 1:**
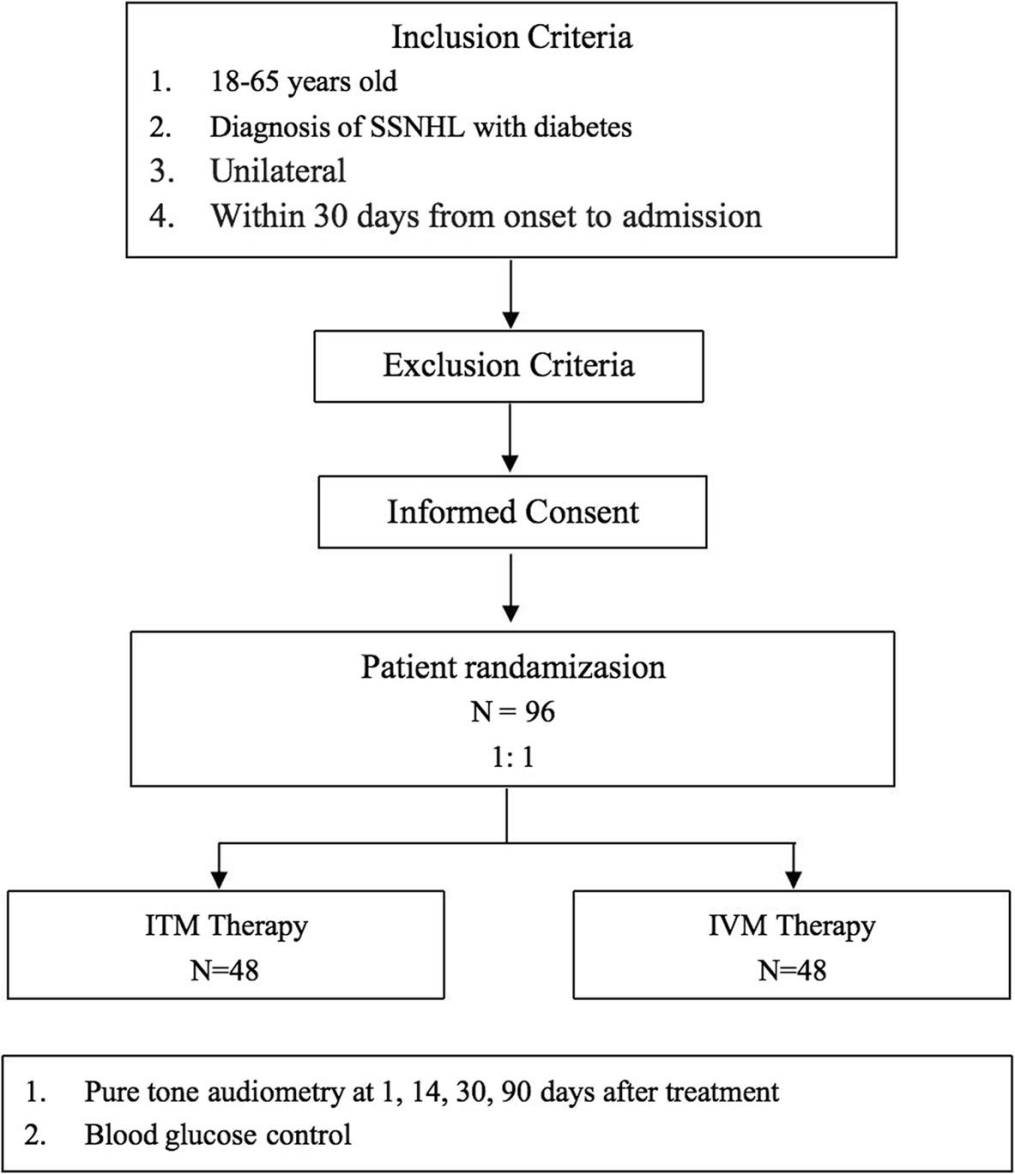
Study flow chart. SSNHL, Sudden sensorineural hearing loss; ITM, intratympanic methylprednisolone; IVM, intravenous methylprednisolone

This study will be a randomized controlled trial that will be performed at the Peking University Shenzhen Hospital in China. We will enroll 86 patients with diabetes mellitus and SSNHL over a 3-year period. Study participants will be randomized at a ratio of 1:1 to receive either intratympanic methylprednisolone or intravenous methylprednisolone (Fig. 1). The study protocol will follow the Standard protocol items: recommendation for interventional trials (SPIRIT) recommendations for interventional trials (Additional file 1).

After receiving written informed consents, patients will be randomly allocated to one of two groups to undergo treatment for 10 days. The follow-up period will be 90 days. This trial protocol was approved by the ethics committees of Peking University Shenzhen Hospital and registered at www.chictr.org.cn on 2 May 2018 (ChiCTR1800015954) under the principal investigator Weiqiang Yang.

### Study participants

Patients with diabetes mellitus and a clinical diagnosis of SSNHL will be enrolled into this study. Based on the Clinical Practice Guidelines of the Sudden Hearing Loss of American Academy of Otolaryngology, Head and Neck Surgery Foundation in 2012, SSNHL is defined as a sensory neural hearing loss greater than 30 dB across three contiguous pure-tone frequencies occurring within 72 h [1]. The criteria for the diagnosis of diabetes mellitus is compliant with the Standards of Medical Care in Diabetes-2010 of the American Diabetes Association [13].

Research assistants will introduce and discuss the trial contents with all potential study participants using Mandarin or the local language. After screening, all eligible subjects will be required to sign a consent form and will be provided information on the main aspects of the trial. Patients will then be able to have an informed discussion with their family and participating consultant. Research assistants will then collect signed consent forms from patients willing to participate in the trial.

#### Recruitment procedure

The majority of the study participants will be recruited through posters on the Internet and in hospitals. Recruitment will also be offered to patients via the trial center.

#### Inclusion criteria

Patients will be enrolled if they have a clinical diagnosis of SSNHL with diabetes mellitus. SSNHL is defined as a sensory neural hearing loss greater than 30 dB across three contiguous pure-tone frequencies of pure tone audiometry occurring within 72 h. A diagnosis of diabetes mellitus will be based on the Standards of Medical Care in Diabetes-2010 [13]. Pure-tone average (PTA) will be calculated using the arithmetic mean of the hearing thresholds at 500, 1000, 2000, and 4000 Hz in the affected ear. Patients must have unilateral SSNHL. Study participants mus be between 18 and 65 years old. The time from onset to admission must be within 30 days and the hearing loss must have been deemed idiopathic following a suitable otolaryngologic evaluation. This will include medical and otologic history and extensive systems review, head and neck otologic and neurotologic physical examination, audiometry, and imaging to rule out structural or retrocochlear conditions, such as vestibular schwannoma, stroke, or demyelinating disease.

#### Exclusion criteria

The exclusion criteria are:

1. External ear inflammation, tympanitis, or external auditory canal swelling in the ears;
2. Diagnosis of Meniere’s disease in the affected ear, chronic suppurative otitis media or cholesteatoma of the middle ear, or ear sclerosis;
3. History of congenital hearing loss;
4. History of ear barometric injury or trauma;
5. History of drug-induced or syphilitic deafness;
6. Diabetes mellitus combined with history of diabetic foot, diabetic nephropathy, diabetic retinopathy, cerebral hemorrhage, or cerebrovascular embolism;
7. Diabetes mellitus combined with active-phase tuberculosis, rheumatic disease, severe coronary atherosclerosis, severe psychosis, or pancreatitis;
8. Chronic renal insufficiency, severe osteoporosis, alcoholism, head and neck malignant tumor, or history of radiotherapy and chemotherapy;
9. Combined immunodeficiency virus, hepatitis B virus, hepatitis C virus, or herpes zoster virus infection;
10. Use of chemotherapy or immunosuppressant drugs within the first 2 weeks;
11. Allergy to the trial drugs.

### Interventions

Participants in the study group will receive four 1-mL doses of 40 mg/mL of methylprednisolone over 1 week, with a dose administered every 2 days by tympanic membrane injection into the middle ear by an otolaryngologist using an operating microscope: 2% tetracaine solution will be used for local anesthesia. Patients will be in the supine position with the affected ear slightly raised and will remain in this position for 30 min after administration of the injection. Patients will also be required to avoid swallowing and vocalization during the treatment period. Patients in the control group will receive a 1-week treatment with intravenous methylprednisolone (1 mg/kg/day; the maximal dose is 60 mg/day) for 5 days, and then have 2 days off. Good Manufacturing Practice (GMP)-grade methylprednisolone will be purchased from Pfizer Manufacturing, Belgium NV.

#### Rescue and concomitant treatment

All study participants will be hospitalized. During the duration of their treatment, patients will be on a low-salt diabetic diet, and receive medications, including mecobalamine (Eisai China Co., Ltd., 0.5 mg three times per day) and extract of Ginkgo Bilobaleaves (Chi Sheng Pharma & Biotech Co., Ltd., Taiwan, China, 70 mg per day). Glycemic levels will be closely monitored and insulin will be administered if necessary. For patients with serious vertigo after tympanic injection, diphenhydramine (20-mg intramuscular injection per episode) will be used as the rescue drug following the physician’s advice.

### Randomization and blinding

Study participants will be randomly allocated to one of two different treatment groups at a 1:1 ratio using the SAS9.2 software with block randomization; block = 4 or 6. Randomization will be concealed until interventions are assigned and enrollment, follow up, data collection, data cleaning, and analysis are performed. It will not be possible to blind the study participants, physicians and paramedics. The investigators, outcomes assessors, and statisticians will be blinded to treatment allocation, and the allocation will only be revealed at the end of study. The randomization list and blinding codes will be kept strictly confidential.

### Outcome measures

The primary outcome measure will be the change in PTA (dB) from the first audiogram to the 30-day follow-up audiogram. Hearing will be evaluated using air-conducted and bone-conducted PTA after 1 and 14 days of treatment, and at 30 and 90 days of follow up.

**Fig. 2:**
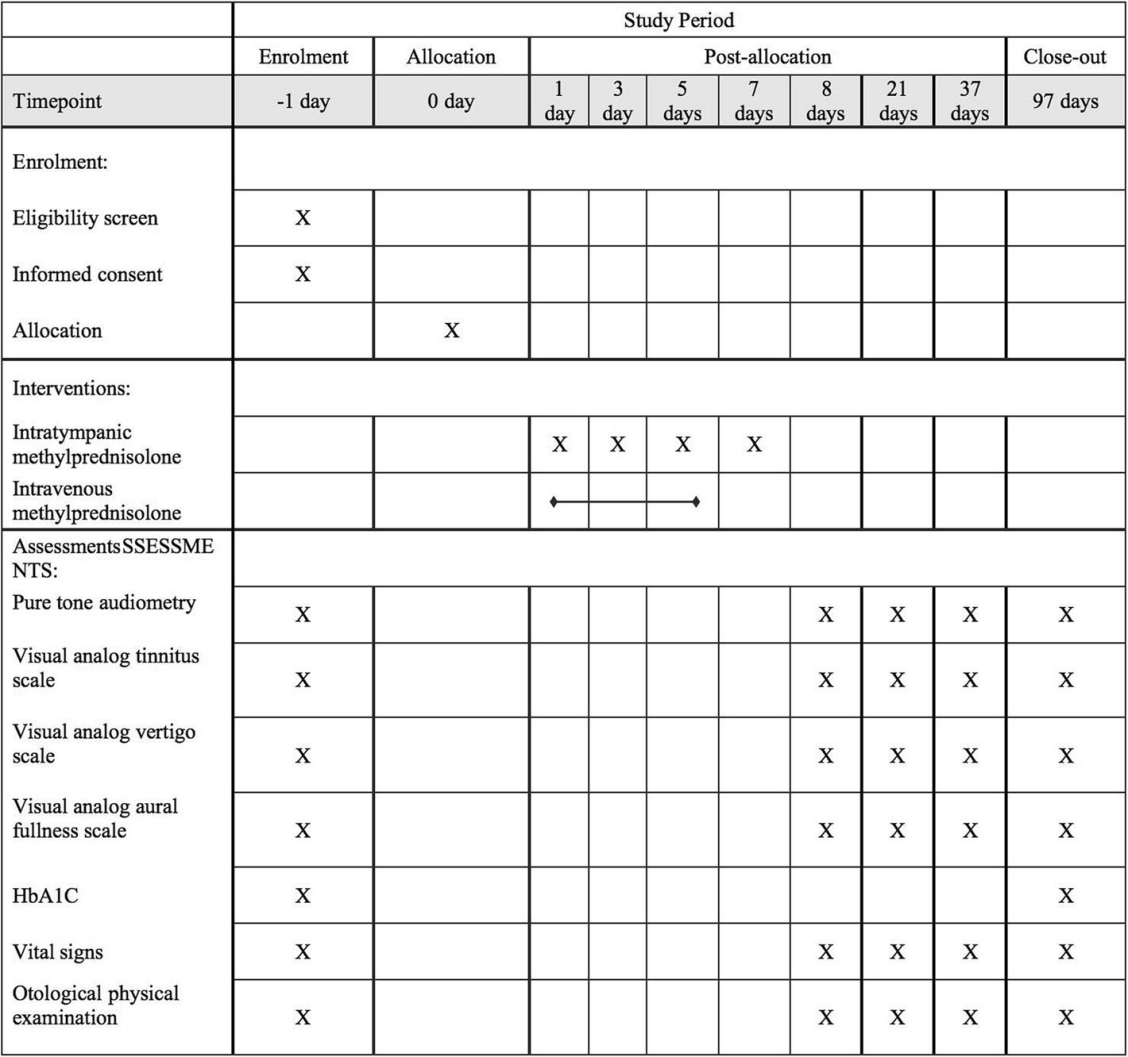
Schedule of study events. HbA1C, glycosylated hemoglobin

Secondary outcome measures will include PTA at 90 days, visual analog tinnitus scale, visual analog vertigo scale, visual analog aural fullness scale, fasting blood glucose and 2-h postprandial blood glucose during treatment, and the change in glycosylated hemoglobin (HbA1C) levels. Vital signs and otological physical examination will be performed at each follow-up visit (Fig. 2).

### Safety assessments

Study participants will be requested to report all adverse events (AEs) at each follow-up visit. All AE reports will be recorded and assessed by the investigators. Blood test, urinalysis, hemagglutination test, and electrocardiograph examinations will be performed before and after the treatment. All abnormal changes from baseline laboratory test results will be evaluated. Other safety test parameters will be measured at the discretion of the treating physician and will be based on the patient’s medical history.

### Sample size calculation

The purpose of the proposed study is to compare the effectiveness of PTA by one-sided *t* test, which is used with the 95% confidence interval method. The difference in PTA is reported in previous publications as 30.7 in the experimental group and as 28.7 in the control group. The difference in the mean was 2, and the combined standard deviation was 3. The type 1 error was 2.5% and statistical power was 90%. Considering a 5% loss rate, 96 samples are required. This allows for 48 participants in the study group and 48 in the control group.

### Data management and quality control

Data collected in this trial will be derived from case reports. The collected data will be entered using the double-entry method at the end of every patient visit.

To ensure a high standard of outcome assessment in accordance with the trial protocol, physicians, assessors, and research assistants will attend a 5-h training workshop before the commencement of the trial. They will also be provided with a written protocol and standard operation procedures. All data gathered from different sites will be verified regularly by a researcher from the Peking University Shenzhen Hospital and will be overseen by study monitors. The monitoring tasks for the trial will be entrusted to the Clinical Research Center of Guangzhou University of Chinese Medicine (Guangzhou, China).

Study participants may withdraw from the study for any reason at any time. If a patient wishes to quit the study, the clinician will ask if they are willing to complete the evaluation based on the research schedule and record the last period during which they took their medication. The incidence of loss to follow up or withdrawal of participants will be recorded and reported.

### Statistical analysis

The primary study hypothesis is that intratympanic methylprednisolone is superior to systemic methylprednisolone for the treatment of hearing loss. Based on a previous study [12], we will define intratympanic treatment as superior if the mean post-treatment change in dB PTA in the systemic group exceeds that in the intratympanic group. The primary outcome, i.e., change in PTA, will be calculated as the difference between the baseline and 30-day follow-up visit. This will then be compared between the two groups using a one-sided *t* test. The two groups will be compared using the 95% confidence interval method to determine superiority.

Data will be statistically analyzed using PASW Statistics 18.0 (IBM SPSS Inc., Armonk, New York, USA) and SAS 9.2 software (SAS Institute Inc., Cary, USA). For secondary outcomes, a two-tailed *P* value < 0.05 will be considered statistically significant. The analysis will be based on the thematic intention-to-treat principles.

We will evaluate safety by tabulating AEs using descriptive statistics at baseline and follow-up visits for each group. Fisher’s exact test and the chi-square test will be used to compare categorical variables between the two groups.

## Discussion

Corticosteroids are considered to be the primary treatment strategy for SSNHL; however, the specific mechanism of action is unknown [14, 15]. Routes of administration include the systemic intravenous or oral route and the topical route via the round window by intratympanic injection. They are both used to increase the concentration of corticosteroids in the inner ear [8]. The therapeutic strategy of using systemic corticosteroids was based on a randomized, placebo-controlled trial performed in 67 patients that showed significantly higher rates of improvement in the corticosteroid-treated group [16]. The study demonstrated improved hearing recovery in 32% (11 of 34) of study participants in the placebo group and in 61% (20 of 33) in the steroid-treated group. Since then, this treatment strategy has been widely used. However, the effectiveness of systemic corticosteroids is still controversial [17]. A Cochrane review that was first published in 2006 and updated in 2013, found that only three trials that met their inclusion criteria with 267 study participants [18]. Two of the three trials demonstrated the lack of efficacy of systemic corticosteroids to improve hearing compared to the placebo control group. The authors concluded that the efficacy of systemic corticosteroids for the treatment of SSNHL remained uncertain. This was because the randomized controlled trials had contradictory outcomes.

Systemic corticosteroid therapy may induce potential adverse effects in several organ systems, in addition, patients may suffer from insomnia, dizziness, weight gain, increased sweating, gastritis, mood changes, photosensitivity, hyperglycemia, pancreatitis, bleeding, hypertension, cataracts, myopathy, opportunistic infections, osteoporosis, osteonecrosis manifesting as fractures, and aseptic necrosis of the femoral and humeral heads [1]. Systemic corticosteroids enhance muscle protein breakdown, adipose tissue lipolysis, and hepatic gluconeogenesis, and reduce glucose utilization, which results in hyperglycemia [19]. Corticosteroid-induced hyperglycemia requires close attention in patients with diabetes mellitus. Moreover, even short-term systemic corticosteroid administration in patients with diabetes mellitus may increase the risk of fractures, venous thromboembolism, and hospitalization for sepsis [20]. Hence, clinicians should be aware of the effects and risk of systemic corticosteroids, especially when treating patients with diabetes mellitus.

Intratympanic corticosteroid treatment is an alternative primary treatment option for patients with diabetes mellitus and SSNHL. Intratympanic administration of corticosteroids provides a direct route into the inner ear via the round window membrane, while systemic corticosteroids have a limited ability to reach the inner ear due to the blood–labyrinthine barrier [21]. Intratympanic corticosteroid administration has been shown to achieve higher concentration levels in the inner ear compared to systemic administration, in both animal and human studies [11, 22]. Several studies have shown that intratympanic corticosteroid administration is as effective as systemic corticosteroids for hearing improvement [12, 23, 24]. In 2011, Rauch et al. [12] performed a multicenter, prospective randomized controlled trial comparing the outcomes of primary treatment with oral prednisone and intratympanic methylprednisolone administration. They observed a mean PTA improvement of 30.7 dB in the oral prednisone group compared to 28.7 dB improvement in the intratympanic group. They dismissed the idea that intratympanic administration was inferior to that of oral administration.

Diabetes mellitus is a risk factor and a poor prognostic factor for SSNHL. A nationwide epidemiological survey based on 3419 patients in Japan showed that 17% of patients with SSHNL had diabetes mellitus and these patients had more severe hearing loss compared to patients who did not have diabetes mellitus [25]. In addition, several studies have shown that intratympanic corticosteroid treatment is as effective as systemic corticosteroid administration in patients with diabetes mellitus and SSHNL. Kakehata et al. [9] performed a prospective, non-randomized, comparative study of 31 patients to evaluate the efficacy of these two different types of corticosteroid administration in patients with diabetes mellitus and SSNHL. There were no significant differences between the two groups in cure rates and successful treatment. In another prospective, non-randomized clinical trial, 114 patients with diabetes mellitus and SSNHL were assigned to one of three groups: a peroral group (48 patients), an intravenous group (32 patients) and an intratympanic group (34 patients). At 8 weeks, there was a statistically significant hearing improvement before and after treatment in each group, but no significant differences were observed in hearing gain among the three groups. One patient in the peroral group and two patients in the intravenous group had uncontrolled hyperglycemia. Two patients in the intratympanic group had perforations of the tympanic membrane. However, the study quality was poor, with different corticosteroids used in the different groups. Patients with diabetes mellitus and SSNHL may benefit more when administered intratympanic corticosteroids; however, the advantage of intratympanic corticosteroids over systemic corticosteroids these patients has not been demonstrated in randomized controlled trials.

A pilot study was necessary to demonstrate the feasibility of this trial. This was performed to inform the design and was conducted from March 2018 to February 2019 in Shenzhen, China. The feasibility trail used the same prospective, randomized controlled trial to compare intratympanic corticosteroid treatment to systemic corticosteroid in patients with diabetes mellitus and SSNHL. The results demonstrated hearing improvement in the intratympanic corticosteroid group (7 patients) of 40 dB versus 30 dB in the systemic corticosteroid group. While the results supported our sample size calculation, we revised a few details in the study design for patient recruitment and trial initiation.

As a prospective, randomized, superiority trial, it is unusual that both the intratympanic and intravenous methylprednisolone group receives adjuvant therapy. We aim to conduct this trial to demonstrate both the superior efficacy and safety of intratympanic methylprednisolone over intravenous methylprednisolone for patients with diabetes mellitus and SSNHL; this trial should be able to determine which corticosteroid administration route will benefit these patients. In addition, high-quality data will be generated to determine whether intratympanic corticosteroid treatment is safe and efficacious in patients with diabetes mellitus and SSNHL.

### Trial status

Protocol version 1.0; 22 September 2017. Recruitment began on 1 March 2018 and will be completed by June 2022.

## Supplementary information

**Additional file 1.** SPIRIT 2013 checklist: recommended items to address in a clinical trial protocol and related documents.

## Data Availability

Not applicable.

## Abbreviations

HbA1C: Glycosylated hemoglobin
PTA: Pure-tone average
SSNHL: Sudden sensorineural hearing loss
